# *BCR-ABL1-* positive chronic myeloid leukemia with erythrocytosis presenting as polycythemia vera: a case report

**DOI:** 10.1186/1752-1947-9-30

**Published:** 2015-04-08

**Authors:** Mihaela I Precup Cornea, Emmanuel Levrat, Paul Pugin, Daniel C Betticher

**Affiliations:** Hôpital Cantonal Fribourg, Chemin des Pensionnats 2, 1708 Fribourg, Switzerland

**Keywords:** Erythrocytosis, Polycythemia vera, Chronic myeloid leukemia, *BCR-ABL1*, *JAK2 V617F*

## Abstract

**Introduction:**

The World Health Organization classification of chronic myeloproliferative disease encompasses eight entities of bone marrow neoplasms, among them Breakpoint cluster region-Abelson murine leukemia viral oncogene homolog 1-positive chronic myeloid leukemia and polycythemia vera. Polycythemia vera requires, in the majority of cases (95%), the negativity of Breakpoint cluster region-Abelson murine leukemia viral oncogene homolog 1 rearrangement and the presence of the Janus kinase 2 mutation. We report a case of erythrocytosis as the primary manifestation of a chronic myeloid leukemia, with the presence of the Philadelphia chromosome and the Breakpoint cluster region-Abelson murine leukemia viral oncogene homolog 1 fusion gene, and in the absence of any Janus kinase 2 mutation.

**Case presentation:**

A 68-year-old Caucasian woman, with a history of cigarette consumption and obstructive sleep apnoea syndrome (undergoing continuous positive airway pressure treatment) had presented to our institution with fatigue and a hemoglobin level of 18.6g/L, with slight leukocytosis at 16G/L, and no other anomalies on her complete blood cell count. Examination of her arterial blood gases found only a slight hypoxemia; erythropoietin and ferritin levels were very low and could not explain a secondary erythrocytosis.

Further analyses revealed the absence of any Janus kinase 2 mutation, thus excluding polycythemia vera. Taken together with a high vitamin B12 level, we conducted a Breakpoint cluster region-Abelson murine leukemia viral oncogene homolog 1 gene analysis and bone marrow cytogenetic analysis, both of which returned positive, leading to the diagnosis of chronic myeloid leukemia.

**Conclusions:**

To date, this case is the first description of a Breakpoint cluster region-Abelson murine leukemia viral oncogene homolog 1-positive chronic myeloid leukemia, presenting with erythrocytosis as the initial manifestation, and mimicking a Janus kinase 2 *V617F*-negative polycythemia vera. Her impressive response to imatinib therapy underscores the importance of not missing this diagnosis.

## Introduction

The World Health Organization 2008 classification of myeloproliferative neoplasms (MPN) encompasses breakpoint cluster region-Abelson murine leukemia viral oncogene homolog 1 (*BCR-ABL1*)-positive chronic myeloid leukemia (CML) and 7 Philadelphia-negative (Ph negative) MPN.

The *BCR-ABL1* fusion gene is consistently found in CML. Conversely, Janus kinase 2 (*JAK2*) mutations, especially *JAK2 V617F*, are frequently encountered in Ph negative MPN, particularly in polycythemia vera (>95%) [1–3], primary myelofibrosis (50%) [3], and in essential thrombocythemia (40 to 50%) [1]. Other gain of function mutations in key proliferative genes such as *MPL W515K/L* and, recently, in the calreticulin (*CALR*) gene, have also been described in Ph negative MPN [4]. Although Ph negative MPN requires evidence of absence of *BCR-ABL1*, CML may rarely harbor *JAK2* mutations, especially during evolution of the disease [4].

Neutrophilic leukocytosis is the initial major finding of CML, which is defined by the World Health Organization as a MPN that originates in an abnormal pluripotent bone marrow stem cell, with the *BCR-ABL1* fusion gene found in all myeloid lineages, as well as in some lymphoid and endothelial cells [2, 3].

Erythrocytosis is usually the main feature of polycythemia vera and is considered, with one of the *JAK2* mutations, as a major criteria of this Ph negative MPN. However, to our knowledge, it has never been reported so far as a manifestation of CML. In our case report, we describe a patient with isolated erythrocytosis as the sole initial manifestation of *BCR-ABL1*-positive CML.

## Case presentation

A 68-year-old Caucasian woman, with active tabagism (40 pack-years), obstructive sleep apnoea syndrome under continuous positive airway pressure (CPAP) via nasal mask, and hypothyroidism secondary to a partial thyroidectomy in 1979, was investigated by her practitioner for fatigue in 2010. The current treatment at that time was aspirin 100mg per day, levothyroxine 100μg per day, and lorazepam 1mg per day. Her family history was negative for any hematologic malignancy.

A hemoglobin (Hb) level of 177g/L and hematocrit (HCT) level of 55% was found, with slight leukocytosis at 12.3G/L, but there was no other abnormality found on her blood smear, especially no left deviation or myelemia. Therefore it was initially interpreted as secondary erythrocytosis.

In 2012 a rise in her Hb level was noted and she was seen at our institution for further investigation. Her test results indicated a Hb level of 180g/L, a HCT level of 55%, a leukocytes count of 16G/L with normal differentiation, a thrombocytes count of 294G/L, and a reticulocytes count of 67G/L. Her arterial blood gas levels were: partial pressure of oxygen in the arterial blood (PaO2) of 8.8kPa, partial pressure of carbon dioxide in the arterial blood (PaCO2) of 4.7KPa, carboxyhemoglobin at 8.6%, a pH of 7.42, and a base excess of -1.2mmol/L with oxygen saturation at 95%. Her lactate dehydrogenase value was 527U/L (normal value: <450U/L), with her liver test results showing normal function. Her erythropoietin and ferritin values were low at 2.3U/L (normal value: 8 to 22U/L) and 16μg/L (normal value: 30 to 300μg/L), respectively. In this context, polycythemia vera was suspected, but could not be confirmed by molecular biology analysis as she was negative for the *JAK2 V617F* mutation. Interestingly, her vitamin B12 level was very high at 1,703pg/mL (normal value: 185 to 1,060pg/mL).

Her blood marrow biopsy revealed a slight hypercellularity with discrete hyperplasia and slight atypia of megakaryopoiesis, normal erythropoiesis with discrete signs of dyserythropoiesis without hyperplasia, and normal myelopoiesis. These abnormalities were aspecific and not suggestive for a MPN. Based on hyperleukocytosis and a high vitamin B12 level, we searched for the *BCR-ABL1* fusion gene, which was surprisingly positive. Her bone marrow conventional cytogenetic analysis revealed a female karyotype with the presence of the Philadelphia chromosome (t (9; 22) (q34; q11.2)) in 85% of metaphases.

An interphase fluorescence in situ hybridization (FISH) analysis confirmed the *BCR-ABL1* rearrangement in 88.5% of interphases analyzed. In light of these results we diagnosed a *BCR-ABL1*-positive CML, in chronic phase, with a low risk Sokal score (0.62). Given the presence of the Philadelphia chromosome (t (9; 22) (q34; q11.2)), we did not search for exon 12 mutations.

**Figure 1:**
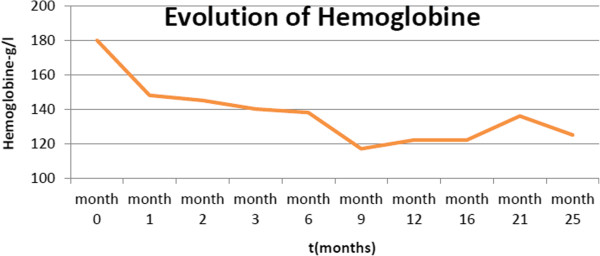
Evolution of hemoglobin under imatinib mesylate therapy.

**Figure 2:**
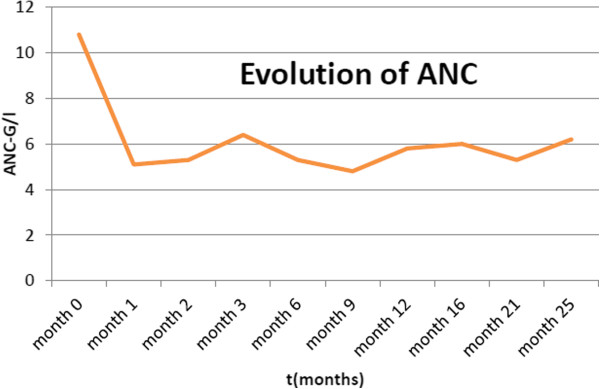
Evolution of absolute neutrophil count (ANC) under imatinib mesylate therapy.

**Figure 3:**
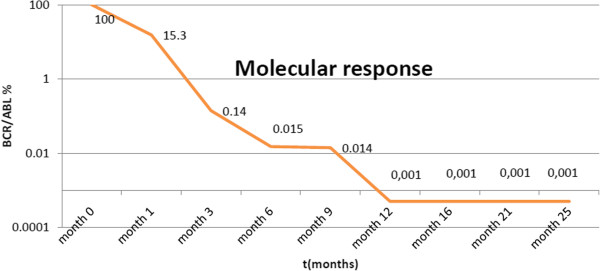
Molecular response under imatinib mesylate therapy. BCR/ABL, Breakpoint cluster region-Abelson murine leukemia viral oncogene homolog 1.

We started a therapy of imatinib mesylate (daily dose of 400mg) on 13 April 2012, and she showed relatively good tolerance. Under treatment, she obtained a complete hematological response at three months, including rapid disappearance of her erythrocytosis (see Figure 1), normalization of absolute neutrophil count (see Figure 2) with complete cytogenetic response and major molecular responses being observed at six months (see Figure 3). These responses were considered optimal according to European LeukemiaNet criteria 2010 and 2013 [5].

## Discussion

In 1951, William Dameshek, described the first concept of myeloproliferative disorders defining five entities of stem cell diseases: CML, polycythemia vera, essential thrombocythemia, primary myelofibrosis, and erythroleukemia [6]. More than five decades later, the revised 2008 World Health Organization classification [2] of MPN encompasses eight entities, among them CML harboring the *BCR-ABL1* translocation and polycythemia vera, which both share a common stem cell-derived clonal heritage, but different phenotypes (leukocytosis with myelemia in CML and polycythemia in polycythemia vera). These alterations result from an abnormal signal transduction, secondary to mutations in genes encoding tyrosine kinase proteins or related molecules (*BCR-ABL1* fusion gene in CML and *JAK2 V617F* mutation in polycythemia vera) [7].

In polycythemia vera, polycythemia is defined as an increased mass of red blood cells and can be evaluated by laboratory findings (Hb or HCT >99th percentile of method-specific reference range for age, sex, and altitude of residence) [2] or measurement of red cell volume by radioisotopic methods, allowing to differentiate between true polycythemia, as in polycythemia vera, chronic obstructive pulmonary disease, and paraneoplastic syndromes, and pseudoerythrocytosis found in a dehydration state, for example.

Several diagnostic algorithms of newly diagnosed (acquired) erythrocytosis investigation are based on erythropoietin levels to differentiate between primary (low erythropoietin levels) with gene alterations and secondary (high erythropoietin levels) erythrocytosis.

In our patient who smoked and was diagnosed with sleep apnea, low erythropoietin levels prevented us from trivializing her polycythemia and misdiagnosing a hypoxemia-related erythrocytosis. Therefore, our report here emphasizes the importance of erythropoietin level measurement in all polyglobulic patients. We agree with the algorithms of Tefferi *et al*. [3] and Spivak and Silver [8] recommending erythropoietin level measurement and searching for the *JAK2 V617F* mutation.

Other nonspecific markers are helpful in diagnosing MPN, such as splenomegaly (absent here), high lactate dehydrogenase, and high vitamin B12 levels, as in our patient. Furthermore, low ferritin levels can contribute to the diagnosis of primary polycythemia, reflecting high iron consumption by increased erythropoiesis.

In the presence of primary polycythemia, further investigations are indicated [3]: bone marrow analysis, showing panmyelosis (hypercellularity with tri-lineage growth) in polycythemia vera; bone marrow cytogenetic analysis (presence of the Philadelphia chromosome in CML); and molecular analysis (presence of the *JAK2 V617F* mutation in polycythemia vera and the *BCR-ABL1* fusion gene in CML). In fact, there have been some rare case reports on patients harboring both the *BCR-ABL1* fusion gene, and the *JAK2 V617F* mutation. However, the co-existence of both mutations appeared during the evolution of the disease [9].

To the best of our knowledge, this is the first case reporting primary polycythemia associated with *JAK2 V617F* mutation negativity, but with the presence of the *BCR-ABL1* fusion gene, pathognomonic of a CML diagnosis. We therefore conclude based on the low erythropoietin level and the *BCR-ABL1* positivity, supporting the monoclonality of red stem cells, as well as the excellent evolution of erythrocytosis under imatinib therapy, that polycythemia without a high number of neutrophils may also be a feature of CML.

## Conclusions

To the best of our knowledge, this case is the first description of a CML with erythrocytosis as the initial manifestation, mimicking a *JAK2 V617F*-negative polycythemia vera.

In such cases we propose therefore to always test not only for the *JAK2 V617F* mutation or exon 12 mutations, but also for the *BCR-ABL1* fusion transcript and to perform bone marrow cytogenetic analysis.

## Consent

Written informed consent was obtained from the patient for publication of this case report and any accompanying images. A copy of the written consent is available for review by the Editor-in-Chief of this journal.

## Abbreviations

*BCR-ABL1*: Breakpoint cluster region-Abelson murine leukemia viral oncogene homolog 1
*CALR*: Calreticulin
CML: Chronic Myeloid Leukemia
Hb: Hemoglobin
HCT: Hematocrit
*JAK2*: Janus kinase 2
MPN: Myeloproliferative Neoplasms
PaCO2: Partial pressure of carbon dioxide in the arterial blood
PaO2: Partial pressure of oxygen in the arterial blood.
